# Design and Optimization of a BAW Microphone Sensor

**DOI:** 10.3390/mi13060893

**Published:** 2022-06-02

**Authors:** Huihui Guo, Jianbo Li, Tingting Liu, Mingqiang Feng, Yang Gao

**Affiliations:** 1 School of Information Engineering, Southwest University of Science and Technology, Mianyang 621010, China; lijb@mails.swust.edu.cn (J.L.); liutingting@swust.edu.cn (T.L.); qiangmingfeng@mails.swust.edu.cn (M.F.); gaoy@swust.edu.cn (Y.G.); 2 Robot Technology Used for Special Environment Key Laboratory of Sichuan Province, Mianyang 621010, China

**Keywords:** microphone, film bulk acoustic resonator, diaphragm, acoustic measurement

## Abstract

A wind tunnel experiment is an important way and effective method to research the generation mechanism of aerodynamic noise and verify aerodynamic noise reduction technology. Acoustic measurement is an important part of wind tunnel experiments, and the microphone is the core device in an aerodynamic acoustic measurement system. Aiming at the problem of low sound pressure (several Pa) and the small measuring surface of an experimental model in a wind tunnel experiment, a microphone sensor head with high sensitivity and small volume, based on film bulk acoustic resonator (FBAR), is presented and optimized in this work. The FBARs used as a transducer are located at the edge of a diaphragm for sound pressure level detection. A multi-scale and multi-physical field coupling analysis model of the microphone is established. To improve the performance of the microphone, the structural design parameters of the FBAR and the diaphragm are optimized by simulation. The research results show that the microphone has a small size, good sensitivity, and linearity. The sensor head size is less than 1 mm × 1 mm, the sensitivity is about 400 Hz/Pa when the sensor worked at the first-order resonance frequency, and the linearity is better than 1%.

## 1. Introduction

The noise of military aircraft during flight can be a major factor determining its battlefield survivability because the engine and wing of the aircraft produce a large noise. Therefore, it is of great significance to carry out corresponding noise assessments for noise reduction research. A wind tunnel experiment is an important way and effective method to research the generation mechanism of aerodynamic noise and verify the aerodynamic noise reduction technology. The aeroacoustic test is an important part of the wind tunnel experiment to locate noise by obtaining the spatial distribution of the sound spectrum and sound intensity (such as sound pressure level), and the microphone sensor is the core device in a measurement system. Surface mounting requires that the side length of the microphone be much smaller than the local curvature of the airfoil, and the airfoil thickness places size limitations on the size of the microphone package [1]. In the wind tunnel experiment of aircraft, the experimental model size precludes the use of conventional microphones; an alternative to conventional microphones is a MEMS microphone with a small size.

**Figure 1:**
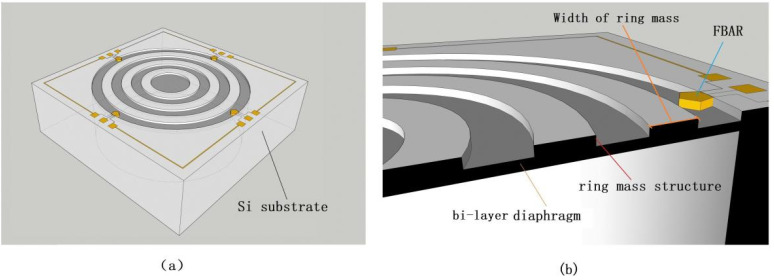
(**a**) Schematic of the proposed microphone head; (**b**) Sectional view of the sensor structure.

In recent years, many researchers have studied MEMS microphones because of their great performance, such as high sensitivities, low power consumption, suitable frequency responses, stability, and reliability, and several MEMS microphones have been developed including capacitance type [2,3,4,5], piezoelectric type [6,7,8,9] and piezoresistive type [10,11,12]. For example, Ganji et al. [4] reported a MEMS capacitive microphone using a perforated diaphragm supported by Z-shape arms with a high open sensitivity of 2.46 mV/Pa, and a small size of 0.3 mm × 0.3 mm; Segovia-Fernandez et al. [6] reported a MEMS piezoelectric acoustic sensor with a sensitivity of 0.68 mV/Pa; Rahaman et al. [7] reported a MEMS piezoelectric acoustic sensor with very high signal-to-noise ratio; Lhermet et al. [10] first reported a piezoresistive microphone based on an in-plane deflecting micro-diaphragm and piezoresistive nano-gauges with a small size and high sensitivity of 0.1 mV/Pa. Although those microphones fit well with MEMS technology and provide a good performance, the capacitive microphone has the problem that the capacitive plate is easy to absorb and adhere to [2], and the piezoelectric microphone has the problems of poor anti-interference ability and low signal-to-noise ratio, and the piezoresistive microphone has the problem of low sensitivity [8]. According to the characteristics of low sound pressure (several Pa) and the small measuring surface of the experimental model in the wind tunnel experiment, it is of great significance to develop sensors with smaller volume and higher sensitivity. The film bulk acoustic resonators (FBAR) have been demonstrably promising in pressure sensors [13,14], owing to their extreme sensitivity, miniature sizes which favor easy integration potential with COMS circuits, and easy arraying for multi-channel functioning. The basic configuration of FBAR is a membrane structure consisting of a piezoelectric thin film sandwiched between two electrodes. The membrane can be formed either by etching the Si substrate from the back or the front surface by etching a pre-buried sacrificial layer. The released membrane structure makes FBAR more sensitive to external forces, which then has great potential to realize high sensitivity [15]. Due to the high performance of the FBAR, a microphone sensor head based on a diaphragm embedded with FBAR as a transducer is presented. The schematic drawing of a microphone is shown in Figure 1.

In this work, a circular diaphragm combined with four small FBARs and ring mass was proposed for the sensor head to measure pressure less than 100 Pa. Compared with other pressure sensors [13,14], the ring mass structure incorporated into the diaphragm helps create stress concentration regions (SCRs) and reduces the defection of the membrane to improve sensitivity. The stress distribution characteristic for the proposed diaphragm under pressure are studied in detail by FEM analyses, and the output characteristic of the FBAR under pressure is calculated using the first-principle methods. By the nonlinear optimization of each dimension variable, an optimized microphone head structure was determined, which made it possible to achieve high sensitivity and linearity, simultaneously.

## 2. Design and Optimization of a Microphone Head Structure

Aiming at the problem of low sound pressure (several Pa) and the small measuring surface of the experimental model in the acoustic wind tunnel experiment, a microphone sensor head structure featuring a circular diaphragm with four FBAR is designed. Four FBARs are located at the edge of a diaphragm, which is used to transform the deformation, under pressure, into a measurable output frequency. Moreover, the dorsal cavity provides space for the deformation of the membrane, as shown in Figure 1b. To fabricate the high performance of the microphone sensor head, the diaphragm structure and FBAR structure should be optimized at the same time.

### 2.1. Diaphragm Structure

The thickness of the diaphragm will limit the measurement range and the sensitivity of stress. Combined with the process characteristics of the FBAR, the bi-layer membrane structure, comprising Si_3_N_4_ and SiO_2_ as the diaphragm layer, is presented, owing to the silicon oxide (SiO_2_) film that has the self-stopped characteristic in the Si DRIE process, and it can also improve the temperature stability of the FBAR [16].

**Figure 2:**
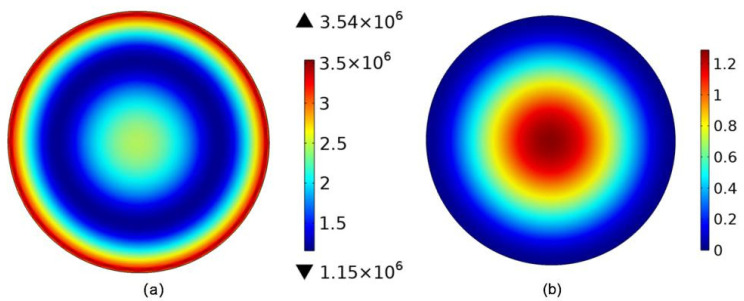
(**a**) The stress distribution of the diaphragm; (**b**) the displacement of the diaphragm.

As the first step to simulations, some estimations about the dimensions of the sensor must be carried out. These calculations, which are based on general structural theories, provide ranges for the main geometrical magnitudes of the device. Louliang et al. [17] discussed the buckling state of the bi-layer diaphragm with different thicknesses of Si_3_N_4_ and SiO_2_, the thickness ratio of the double-layer film shall be greater than two to eliminate the central bending of the diaphragm. Due to our fabrication process constraints, high-quality SiO_2_ film cannot grow too thick on Si substrate. A 0.5 μm SiO_2_ with low stress can be deposited on Si substrate using our equipment. Because the pure SiO_2_ diaphragm suffers from buckling and wrinkling issues due to its internal compressive stress, a 1 μm Si_3_N_4_ layer should be further deposited to counteract membrane internal stress. However, our previous research results show that the resonant frequency of FBAR decreases rapidly with the increase in the Si_3_N_4_ thickness; owing to the high operation frequency that is beneficial in obtaining high sensitivity, the thinner thickness of the Si_3_N_4_ is better [18]. Considering that the Si_3_N_4_ support film has no side effects on the impedance characteristics of the FBAR, the thinnest thickness is preliminarily determined as 0.2 μm. Ignoring the bending of the diaphragm caused by the internal compressive stress of the SiO_2_, the stress distribution and displacement of the diaphragm with 0.2 μm Si_3_N_4_/0.5 μm SiO_2_ under 10 Pa is obtained by the finite element method, as shown in Figure 2. It can be seen that the maximum stress and displacement are 3.6 × 10^6^ Pa and 1.2 μm, respectively. In addition, the maximum strain region of the diaphragm is narrow, which will limit the size design of the sensing element such as FBAR.

**Figure 3:**
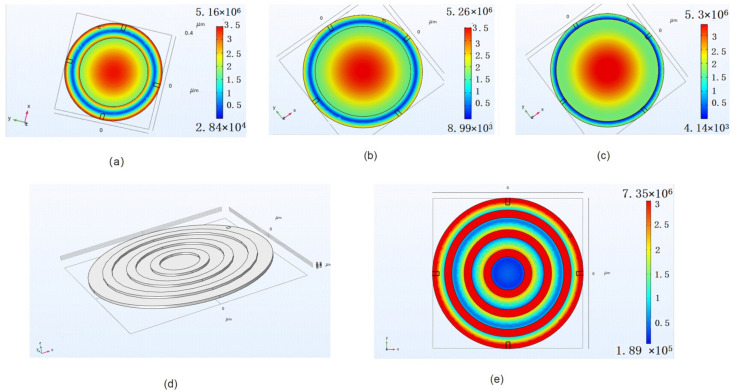
The stress distribution of diaphragm with a single mass structure (**a**) the radius of 280 μm (**b**) the radius of 320 μm (**c**) the radius of 360 μm; (**d**) Three-dimensional FEA model of the diaphragm; (**e**) The stress distribution of diaphragm with a ring mass structure.

To obtain greater stress under the same load, creating stress concentration regions and localizing strain energy within a relatively narrow space is an effective method. A single mass block and a ring mass block are introduced, respectively. A single mass is obtained by increasing the thickness of the Si_3_N_4_ layer. At the same time, the bending of the diaphragm will gradually disappear increasing with the Si_3_N_4_ thickness from 0.2 μm to 0.8 μm. The stress distribution of the diaphragm with different radii of a mass block under 10 Pa is shown in Figure 3. It can be seen that the maximum area is moving toward the edge of a diaphragm with the increasing radius of a mass block, and the maximum strain area is becoming smaller and smaller. Although the maximum strain value has nearly doubled, the increase in average stress in the FBAR area is very small. Compared with the above double-layer structure, the effect of this structure on improving the sensitivity is not obvious. 

**Figure 4:**
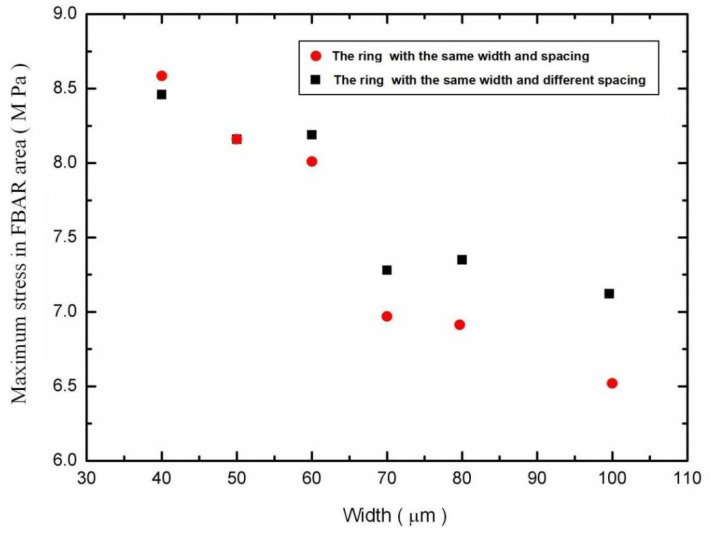
The maximum stress in the FBAR area of the diaphragm with a different ring structure.

Then, the ring mass structure of the diaphragm is designed. The thickness of the annular Si_3_N_4_ and bi-layer diaphragm is 0.4 μm and 0.7 μm (0.2 μm Si_3_N_4_/0.5 μm SiO_2_), respectively. To investigate the relationship between the parameters of the ring structure and the stress distribution of the FBAR area under the same pressure load, the model of the diaphragm is built, as shown in Figure 3d. In this model, the strain distribution in the FBAR area of the diaphragm with different ring parameters under 10 Pa is obtained using the finite element analysis, as shown in Figure 4. It can be seen that the maximum stress increase comes with a decrease in ring spacing. However, the width of the maximum stress area also narrows. Due to the average stress in the FBAR area determining the output of the microphone, the distance between the outer ring and the edge of the film shall not be less than 40 μm. In addition, combined with the process error of diaphragm release and the optimal process size of FBAR in our previous work, the optimal ring structure width and spacing are around 60 μm. Therefore, the preliminary design parameters of the diaphragm structure are shown in Table 1.

Meanwhile, the maximum stress and the displacement of the diaphragm under pressure loads from 0–500 Pa are 0.1 G Pa, which is far less than the fracture strength of the Si_3_N_4_ with ranges 6.9–7.9 G Pa [19]. Therefore, the diaphragm structure is mechanically robust within a range of 0–500 Pa. At the same time, the design sensitivity of the microphone is calculated as 400 Hz/Pa in the full measurement range (0–500 Pa).

### 2.2. FBAR Structure

Although the area of the FBAR will not affect its resonant frequency, the average stress in the FBAR area will determine the output value of the sensor. Therefore, FBAR should be set in the maximum strain region of the film as much as possible. Combined with the above simulation result of the diaphragm, the resonant area of FBAR is set as 30 μm × 30 μm to obtain the maximum strain.

The basic configuration of the FBAR is a membrane structure, consisting of a piezoelectric thin film sandwiched between two electrodes. The membrane can be formed either by etching the Si substrate from the back. The structure parameters of the FBAR are determined by both design considerations and process constraints. In this work, the materials and geometric parameters of the FBAR are shown in Table 2.

**Figure 5:**
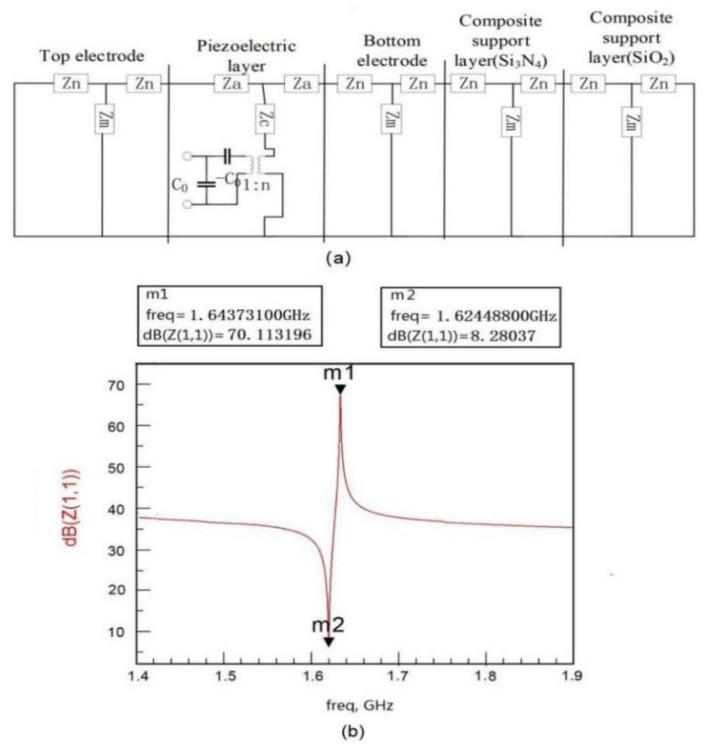
(**a**) Mason model of 5-layer FBAR; (**b**) the impedance characteristic curve of FBAR without pressure load.

The fundamental resonant frequency of FBAR is mainly determined by piezoelectric film, an electrode layer, and a support layer. To obtain the output characteristic of the FBAR under pressure loads, the five-layer FBAR stack is composed of a support layer (bi-layer diaphragm), bottom electrode, piezoelectric layer, and a top electrode is built using the Mason equivalent model (as shown in Figure 5a). With help of the ADS software, the simulated impedance characteristic curve of FBAR without pressure load is shown in Figure 5b. The series resonant frequency of the FBAR marked m 2 is about 1.624488 GHz and the parallel resonant frequency marked with m 1 is about 1.643731 GHz.

**Figure 6:**
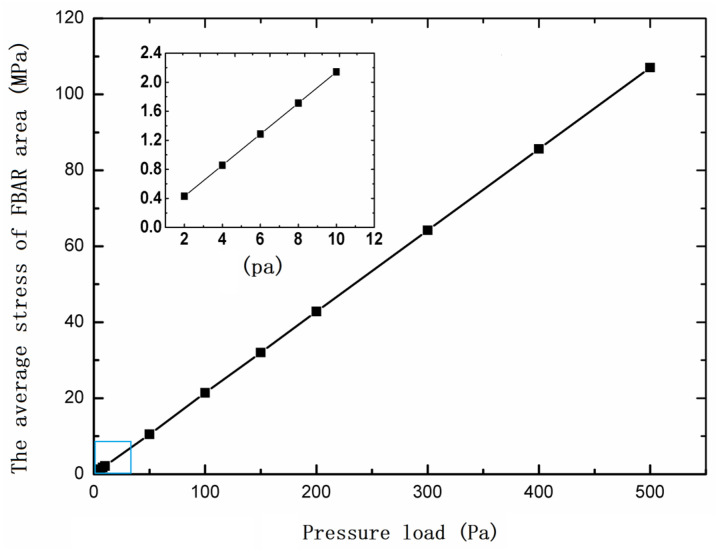
The average stress of FBAR area under different loads; the inset shows the average stress of FBAR area under small loads.

After the position and geometric dimension of FBAR are determined, the average stress of the FBAR area calculated by simulation is shown in Figure 6.

## 3. Output of the Sensor

The FBAR is used as a transducer for sound pressure detection. Therefore, it is very important to analyze and calculate the relationship between stress and FBAR resonant frequency. In this work, the stress in the FBAR area will change the elastic, dielectric, and piezoelectric characteristics of the Aluminum nitride film, and then change the acoustic and electromagnetic characteristics of the FBAR. 

Zhifan Wang et al. [20] performed extensive first-principle studies to discuss the effect of the anisotropic pressure on the physical properties of Aluminum nitride, and found that the pressure-induced variations of the elastic constant, piezoelectric constant, and mass density significantly change the acoustic wave velocity, whereas the effect of static dielectric on acoustic wave velocity is negligible. Then, the elastic constant *C*_33_, piezoelectric constant *e*_33_, mass density ρ, and acoustic wave velocity V of AlN under uniaxial pressure are given respectively as [20]:

(1) $$C_{33}=357.2-2.30P-0.22P^{2}$$

(2) $$e_{33}=1.646+0.079P$$

(3) ρ=3.258+0.006P

(4) $$V=\sqrt{\frac{C_{33}+e_{33}{}^{2}}{\rho }\;}=10,973.36+7.42P-1.42P^{2}$$

where *P* is the uniaxial pressure (G Pa) in the basal plane on AlN film.

**Figure 7:**
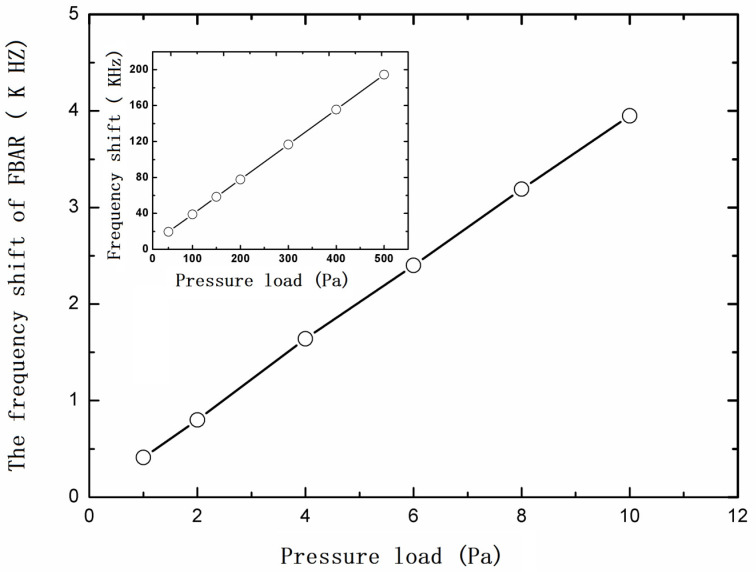
Pressure–Δf characteristic curve of a BAW microphone head in a range from 0–10 Pa; the inset shows the output curve with a large range from 50–500 Pa.

The longitudinal acoustic wave velocity V in the FBAR under different pressure loads can be calculated by Equation (4). With the help of the ADS software, the five-layer Mason equivalent circuit model of the FBAR is established to get the resonant frequency change of the FBAR with the varied longitudinal acoustic wave velocity V in AlN film. The resonant frequency change curve of the sensor under different pressure loads is obtained, as shown in Figure 7. It can be seen that the sensor has good linearity and sensitivity. The linearity is better than 1% in the range from 0 to 10 Pa, and the sensitivity is about 400 Hz/Pa, which can meet the needs of acoustic measurement in wind tunnel experiments. In addition, the inset of Figure 7 shows the output of the sensor in a range up to 500 Pa.

The microphone is usually packaged with a metering structure and a professional signal processing circuit. The performance of the microphone is determined by the sensor head and the signal processing circuit. Using the excellent force sensitivity of FBAR and the design of annular SCRs, a microphone sensor head has achieved a high sensitivity of 400 Hz/Pa and a low non-linearity error of 1% in the range from 0 to 500 Pa. However, the frequency response characteristics and the lower limit of the sound pressure detection of the microphone are determined by the readout circuit. At present, there is no ASIC that can be integrated with the frequency output sensor. The readout circuit of the microphone has been designed based on the pierce oscillation circuit and gap-less counter. This circuit is only suitable for the measurement of low-frequency noise signals, which is limited by the acquisition and processing time of frequency signals. To solve the problem of the frequency response of this kind of microphone, the BAW sensor readout circuit based on a six-port refractometer will be carried out in the future.

## 4. Conclusions

This paper developed a BAW microphone sensor with high sensitivity and a small volume based on a film bulk acoustic resonator for acoustic measurement in wind tunnels. With the help of finite element analysis software, the sensor head structure of the MEMS sensor was optimized. The annular island membrane is presented to obtain more strain energy and reduce the deflection of the diaphragm. Meanwhile, the proposed diaphragm localized more strain energy to improve the sensitivity and enhance linearity. With the excellent force sensitivity of FBAR, the four FBAR are located at the edge of a diaphragm, which is used to transform the stress into a measurable output frequency. Based on the simulation results, the proposed microphone sensor head with a high sensitivity of 400 Hz/Pa, small size of 1 mm × 1 mm, and a low non-linearity error of 1% in the range of 0 to 500 Pa by the geometrical optimization of the annular island membrane structure. Combined with a high-performance readout circuit, this microphone can be used for the noise measurement of low sound pressure of less than 10 Pa in a wind tunnel. 

## Figures and Tables

**Table 1:** The main structural parameters of the diaphragm.

The Radius of the Bi-Layer Diaphragm (μm)	The Thickness of the Bi-Layer Diaphragm (Si_3_N_4_/SiO_2_) (μm)	The Thickness of the Ring Mass Structure (Si_3_N_4_) (μm)	Width and Spacing of Ring Mass Structure (μm)	Number of Ring Mass Structure
400	0.7	0.4	60	3

**Table 2:** Material and geometric parameters of the FBAR.

Material	Density (g/cm^3^)	Dielectric Loss (dB/m)	Acoustic Impedance (kg/m^2^s)	Longitudinal Acoustic Wave Velocity (m/s)	Film Thickness (μm)
SiO_2_	2.3	-	1.25 × 10^7^	6253	0.5
Si_3_N_4_	3.25	-	3.6 × 10^7^	11,000	0.2
Pt	21.45	-	6.0 × 10^7^	2789	0.1
AlN	3.2	800	3.7 × 10^7^	10,984.57	1
Al	2.7	7500	1.76 × 10^7^	6526	0.9

## Data Availability

Data are contained within the article.
